# Chemical disinfection of *Encephalitozoon cuniculi*: toward evidence-based infection control guidelines

**DOI:** 10.3389/fmicb.2025.1744472

**Published:** 2026-01-13

**Authors:** Jianhua Gao, Xianzhi Meng, Zhangshuai He, Yunlin Tang, Jie Chen, Xuemei He, Hua Cao, Shuyan Long, Guoqing Pan

**Affiliations:** 1 State Key Laboratory of Resource Insects, Chongqing Key Laboratory of Microsporidia Infection and Control, Southwest University, Chongqing, China; 2 School of Medical Technology, Jiangxi Medical College, Shangrao, Jiangxi, China; 3 College of Clinical Medicine, Chongqing Three Gorges Medical College, Chongqing, China; 4 College of Sericulture, Textile and Biomass Sciences, Southwest University, Chongqing, China

**Keywords:** chlorine-based agents, disinfection, *Encephalitozoon cuniculi*, flow cytometry, infectivity, murine model, qPCR, sporicidal efficacy

## Abstract

**Background:**

*Encephalitozoon cuniculi* is an opportunistic pathogen with significant zoonotic potential, particularly for immunocompromised individuals. However, evidence-based disinfection protocols against its environmentally resistant spores are lacking, leading to potential reliance on suboptimal agents.

**Objective:**

This study aimed to establish a rigorous, data-driven hierarchy for the efficacy of common chemical disinfectants against *E. cuniculi* spores by integrating assessments of structural integrity, cellular infectivity, and *in vivo* dissemination.

**Methods:**

We employed a multi-modal approach. Spores were treated with rapid-acting (75% ethanol, 1% hydrogen peroxide, chlorine-based agent) and long-acting (nano-silver, quaternary ammonium compounds—QACs) disinfectants. Sporicidal effects were evaluated via flow cytometry using ethidium bromide (EB) staining. Functional infectivity was quantified in Vero cells using a species-specific TaqMan qPCR assay targeting intracellular spore DNA. A murine model was used to assess the capacity of treated spores to establish systemic infection, quantified by spore DNA load in blood and kidney tissue.

**Results:**

Flow cytometry revealed that 75% ethanol caused significant membrane damage (~51% EB-positive spores in 20 min) but failed to consistently abolish infectivity in cell culture or mice. Chlorine-based agents and hydrogen peroxide demonstrated potent, time-dependent inactivation, achieving >96 and 100% infectivity reduction in cells and mice, respectively, within 60 min. Notably, chlorine agents rendered spores non-infectious without inducing proportional EB uptake, indicating a mechanism distinct from gross membrane disruption. The long-acting disinfectant QACs necessitated an extended contact time (up to 5 h) to achieve complete prevention of *in vivo* infection. This prolonged exposure induced substantial spore aggregation, thereby impeding accurate flow cytometric analysis.

**Conclusion:**

The chlorine-based disinfectant and hydrogen peroxide (rapid-acting), as well as the quaternary ammonium compound (long-acting), all effectively eliminated the infectivity of *E. cuniculi* spores. Crucially, we demonstrate a critical dissociation between spore structural damage and viability, highlighting the necessity of functional infectivity assays over structural metrics alone. This study provides a foundational framework for evidence-based infection control guidelines in clinical, veterinary, and public health settings.

## 1 Introduction

*Encephalitozoon cuniculi (E. cuniculi)* is a microsporidian parasite and an emerging opportunistic pathogen with zoonotic potential. It poses significant risks to immunocompromised populations, including HIV/AIDS patients, organ transplant recipients, and individuals undergoing chemotherapy (Mathis et al., 2005; Magalhães et al., 2022). Transmission occurs via environmentally resistant spores shed in urine or feces, which can persist on surfaces and facilitate indirect infection (Didier and Weiss, 2006; Ghebremichael et al., 2022). Despite its clinical relevance, standardized disinfection protocols targeting *E. cuniculi* spores remain underdeveloped (Weber et al., 1994; Didier, 2005). Current guidelines often extrapolate from studies on bacterial or viral pathogens, neglecting the unique resilience of microsporidian spores to chemical agents. For instance, alcohol and sodium hypochlorite—commonly used in clinical and household settings—have shown variable efficacy against *E. cuniculi* in limited studies, with critical gaps in data regarding exposure time and concentration thresholds (Santillana-Hayat et al., 2002; Fayer et al., 2012).

The lack of systematic evaluations is particularly concerning in regions like China, where rising pet ownership increases human-animal contact, amplifying spillover risks (Ding et al., 2017; Li et al., 2019). Existing disinfection practices often rely on anecdotal evidence, risking suboptimal pathogen control. Furthermore, traditional viability assays, such as culture-based methods, may underestimate spore persistence due to residual infectivity undetectable by staining alone (Green et al., 2000). To address these challenges, this study integrates a multi-tiered approach: (1) flow cytometric assessment of spore structural integrity following disinfection (Moss et al., 1999); (2) cell-based infectivity assays and TaqMan qPCR-based detection of intracellular *E. cuniculi* spore DNA post-infection in host cells; and (3) quantification of *E. cuniculi* DNA in blood and kidney at post-infection time points via TaqMan qPCR using murine models infected with spores post-disinfection (Kotková et al., 2018). By evaluating both rapid-acting disinfectants (ethanol, chlorine-based agents, hydrogen peroxide) and long-lasting disinfectants (monomer nano-silver solution, quaternary ammonium compounds), we aim to establish data-driven guidelines for *E. cuniculi* spore inactivation. This work bridges a critical gap in infection control, offering actionable insights for healthcare facilities, veterinary practices, and households.

## 2 Materials and methods

### 2.1 *Encephalitozoon Cuniculi* and cell culture

*Encephalitozoon cuniculi* spores (American Type Culture Collection, Manassas, VA, USA, ATCC #50502) were propagated and harvested from Vero (American Type Culture Collection, ATCC CCL-81™) cells as previously described (Han et al., 2019; de Boer et al., 2021). Briefly, Vero cells were grown in T25 flasks containing Dulbecco’s Modified Eagle Medium (DMEM) supplemented with 10% fetal bovine serum and 1% penicillin–streptomycin, and maintained at 37 °C in a humidified atmosphere of 5% CO₂ (Barrett et al., 2009; Pollock et al., 2020). Cells were then inoculated with spores at approximately 60% confluence at a multiplicity of infection (MOI) of 10 spores per cell (Guidi-Rontani et al., 1999). The culture medium was replaced every 3 days, and amplified spores were harvested after 10–15 days of culture. Spores were collected from the supernatant, purified by filtration through a 3-μm pore-size membrane to remove host cell debris, and enumerated using a hemocytometer prior to use (Han et al., 2019).

### 2.2 Designing of species-specific qPCR primers

Specific primers targeting the internal transcribed spacer (ITS) region of *E. cuniculi* were designed (Ghosh and Weiss, 2009; Santín and Fayer, 2011). The process began with retrieving ITS sequences from various *Encephalitozoon* species from the GenBank database using the NCBI Nucleotide Sequence Search program. Multiple sequence alignments were performed using the Clustal W algorithm within MEGA11 software to identify conserved regions unique to *E. cuniculi*. Candidate primers were subsequently analyzed in silico with Primer-BLAST to assess species specificity. Further evaluation of primer properties, including melting temperature (Tm), potential for dimerization, self-annealing, hairpin formation, and GC content, was conducted using the PrimerSelect module of DNASTAR software (Table 1). Primer specificity was ultimately confirmed by experimental PCR amplification. The templates used for this validation were high-quality genomic DNA from a panel of organisms, including the closely related *Encephalitozoon* species *E. cuniculi* and *E. hellem*, as well as the common unicellular fungus *Saccharomyces cerevisiae* and the prokaryotic bacterium *Escherichia coli*.

**Table 1:** The sequence, target, annealing temperature, and amplicon size of the primers used for qPCR detection.

Primer	Oligonucleotide sequence (5′-3′)	Annealing temperature (°C)	Amplicon size (bp)	Target gene
E.c sense	ATAAGTCGTAACATGGCTGCT	58	82	AJ005581.1
E.c anti sense	ACAAACAAACAAACATCCATCAAA			
E.c probe	CATACTGATCCTGCTGCTGGTTC			

### 2.3 Treatment of spores by various disinfectants

In this experiment, we employed two long-acting sporicidal agents: quaternary ammonium compounds (QACs) and nanosilver disinfectant (Ag). The QAC disinfectant (Brand: Basteur®, Shanghai Basteur Antimicrobial Technology Co., Ltd.) contains alkyl dimethyl benzyl ammonium chloride and dioctyl dimethyl ammonium chloride as its active ingredients, at a concentration of 0.18–0.22% (w/v). The nanosilver disinfectant was an aqueous solution of silver nanoparticles (12–15 nm in diameter, 1,000 ppm), supplied by Shanghai Huzheng Nano Technology Co., Ltd. Three rapid-acting disinfectants were evaluated: 75% ethanol (EtOH), a chlorine-based agent (CL), and hydrogen peroxide (H₂O₂). The chlorine-based disinfectant contained sodium dichloroisocyanurate and trichloroisocyanuric acid as its primary components, with an available chlorine content of 45–55%. The hydrogen peroxide solution had a concentration of 0.9–1.1%. All three rapid-acting disinfectants were supplied by Shanghai Basteur Antimicrobial Technology Co., Ltd.

A spore suspension was prepared at two concentrations in PBS: 2 × 10^7^ spores/mL for Vero cell infection and 2 × 10^8^ spores/mL for mouse intraperitoneal injection. Each 1 mL aliquot of the sample suspension was centrifuged at 8,000 × g for 5 min. The resulting pellet was resuspended in 2 mL of one of the five disinfectants. Samples were exposed to rapid-acting disinfectants for 20, 40, or 60 min, or to long-acting disinfectants for 2, 3, or 5 h. After treatment, the samples were centrifuged, washed with sterile PBS, and stored at 4 °C until subsequent use in cellular or animal experiments.

### 2.4 Validation of sporicidal efficacy at the spore level

In this study, ethidium bromide (EB) (HY-D0021, MCE China) was chosen to assess sporicidal efficacy. EB is a fluorescent dye with a moderate molecular weight of 394.32, which cannot penetrate spores with intact cell walls. However, when sporicidal agents induce structural damage, EB permeates the compromised cells and intercalates with nucleic acids. Under blue light excitation, damaged spores exhibit distinct red fluorescence (Alakomi et al., 2000; Fedorko et al., 2005). Flow cytometry (Guava EasyCyte, MILLIPORE) was utilized to quantify EB staining in spores before and after treatment. The proportion of spores displaying red fluorescence was analyzed to evaluate sporicidal efficacy, enabling precise quantification of agent-induced cellular damage.

### 2.5 Detection of intracellular spore DNA in host cells post-infection

To precisely determine the impact of sporicidal treatments on spore infectivity, spores subjected to different treatments were used to infect Vero cells. At 48 h post-infection, the culture medium was removed from all groups, and extracellular spores were thoroughly washed with PBS. Cells were lysed, and species-specific qPCR using custom-designed TaqMan probes was performed to measure intracellular spores DNA copy number. The sporicidal efficacy was assessed based on the reduction in genomic DNA load, reflecting the ability of treated spores to invade host cells.

### 2.6 Quantification of *Encephalitozoon cuniculi* DNA in murine kidney and blood

Healthy female specific pathogen-free (SPF) C57BL/6 mice (20–22 g) were purchased from Beijing Vital River Laboratory Animal Technology Co., Ltd. (Beijing, China). Spores treated with different sporicidal methods were intraperitoneally injected into the mice. On day 45 post-infection, deep anesthesia was induced in the mice using 5% isoflurane delivered in an oxygen stream within an induction chamber. Once a surgical plane of anesthesia was confirmed (absence of response to toe pinch), the mice were immediately euthanized by cervical dislocation (Elsner et al., 2015; Bodnar et al., 2023). Blood and one kidney were collected from each mouse for *E. cuniculi* DNA quantification by qPCR, while the contralateral kidney was fixed in 4% paraformaldehyde for subsequent fluorescence staining (Sak et al., 2022). For qPCR analysis, blood samples (50 μL) were mixed with 50 μL of one-step lysis buffer (CY-L-001, Shanghai Shuiyuan Biotechnology Co., Ltd.), boiled for 10 min, cooled to room temperature, and stored at −20 °C until use. Similarly, kidney tissue samples (~50 mg) were homogenized in 100 μL of the one-step lysis buffer, boiled for 10 min, cooled to room temperature, and stored at −20 °C. All animal care and experimental procedures were approved by the Institutional Animal Care and Use Committee (IACUC) of Southwest University (approval no.: IACUC-20250304-40).

### 2.7 Fluorescence staining of cells and kidney tissues post-infection with differentially disinfected spores

Mouse kidney tissues were fixed in 4% paraformaldehyde, embedded in paraffin, and sectioned at a thickness of 5 μm. Following deparaffinization and rehydration, the sections were subjected to fluorescence staining. The subsequent staining procedure was nearly identical for both cultured cells and tissue sections. Briefly, samples were fixed with 4% paraformaldehyde for 20 min, washed three times with PBS, and permeabilized with 0.05% Triton X-100 for 25 min. After another PBS wash, the samples were stained with PI (HY-D0815, MCE China) and Calcofluor White (HY-D0367, MCE China) at final concentrations of 10 μg/mL and 1 μg/mL, respectively, and incubated at room temperature for 20 min in the dark (Carriere et al., 2023). Finally, the cell samples were washed three times with PBS and mounted in PBS solution containing 50% glycerol for microscopic observation and imaging. The kidney tissue sections were mounted with a synthetic resin mounting medium for microscopic observation.

### 2.8 Statistical analysis

All experiments were repeated at least three times independently. Data are expressed as the mean ± standard deviation. One-way analysis of variance (ANOVA) was performed to assess significant differences in variables, followed by Tukey’s *post hoc* test (SPSS Statistics v. 23.0; IBM Inc., Chicago, IL, USA). Differences were considered statistically significant at *p* < 0.05.

## 3 Results

### 3.1 Specificity and sensitivity validation of qPCR assay

Using our self-developed species-specific qPCR assay for *E. cuniculi*, we performed absolute quantification of serially diluted *E. cuniculi* genomic DNA. Parallel testing included *E. hellem* (high genomic homology control), yeast genomic DNA (gDNA), and *E. coli* genomic DNA as amplification templates. Results demonstrated a detection limit of 1.4 fg of genomic DNA, with quantifiable spore DNA copy concentrations as low as 1.359 × 10^1^ ± 5.217 copies/μL (*n* = 3). No amplification was detected for *E. hellem* gDNA, yeast gDNA, or *E. coli* genomic DNA (Table 2).

**Table 2:** Mean Ct values and copy numbers of sevenfold serial dilutions of genomic DNA in the qPCR assay.

Genomic DNA	Weight/reaction mix	Ct ± SD (*n* = 3)	copies number ± SD (*n* = 3)
E.c gDNA	14 pg	25.24 ± 0.07	1.425E4 ± 5.782E2
E.c gDNA	1.4 pg	32.27 ± 0.1	2.094E2 ± 1.279E1
E.c gDNA	140 fg	35.41 ± 0.45	3.208E1 ± 9.689E0
E.c gDNA	14 fg	35.94 ± 0.06	2.262E1 ± 8.488E-1
E.c gDNA	1.4 fg	36.88 ± 0.71	1.359E1 ± 5.217E0
E.c gDNA	0.14 fg	N.D.	N.D.
E.h gDNA	11.52 ng	N.D.	N.D.
S.c gDNA	9.35 ng	N.D.	N.D.
E.coli gDNA	39.2 ng	N.D.	N.D.

SD, standard deviation of biological replicates; N.D., Not detected; S.c., *Saccharomyces cerevisiae*, E.h, E. hellem; E.c, *E. cuniculi*.

### 3.2 Flow cytometric analysis of sporicidal efficacy in treated spores

**Figure 1:**
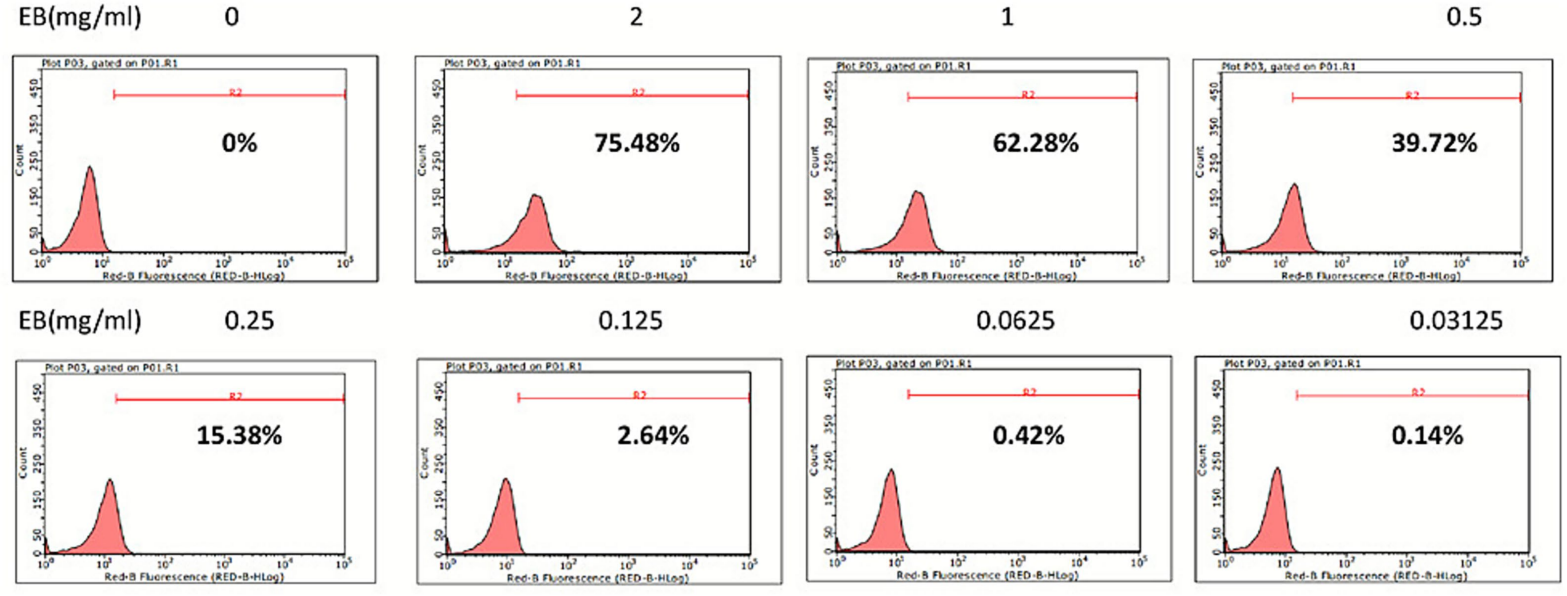
Determination of the optimal EB staining concentration. Exposure to EB concentrations ranging from 2 to 0 mg/mL yielded staining rates of 75.48, 62.28, 39.72, 15.38, 2.64, 0.42, 0.14, and 0%.

To determine the optimal EB concentration for formal experiments, we prepared serially diluted EB solutions and stained untreated *E. cuniculi* spore suspensions of uniform concentration. Staining results revealed that normal *E. cuniculi* spores failed to exhibit fluorescence at concentrations ≤ 0.0625 mg/mL (Figure 1). Consequently, 0.0625 mg/mL was selected for subsequent flow cytometric analysis.

**Figure 2:**
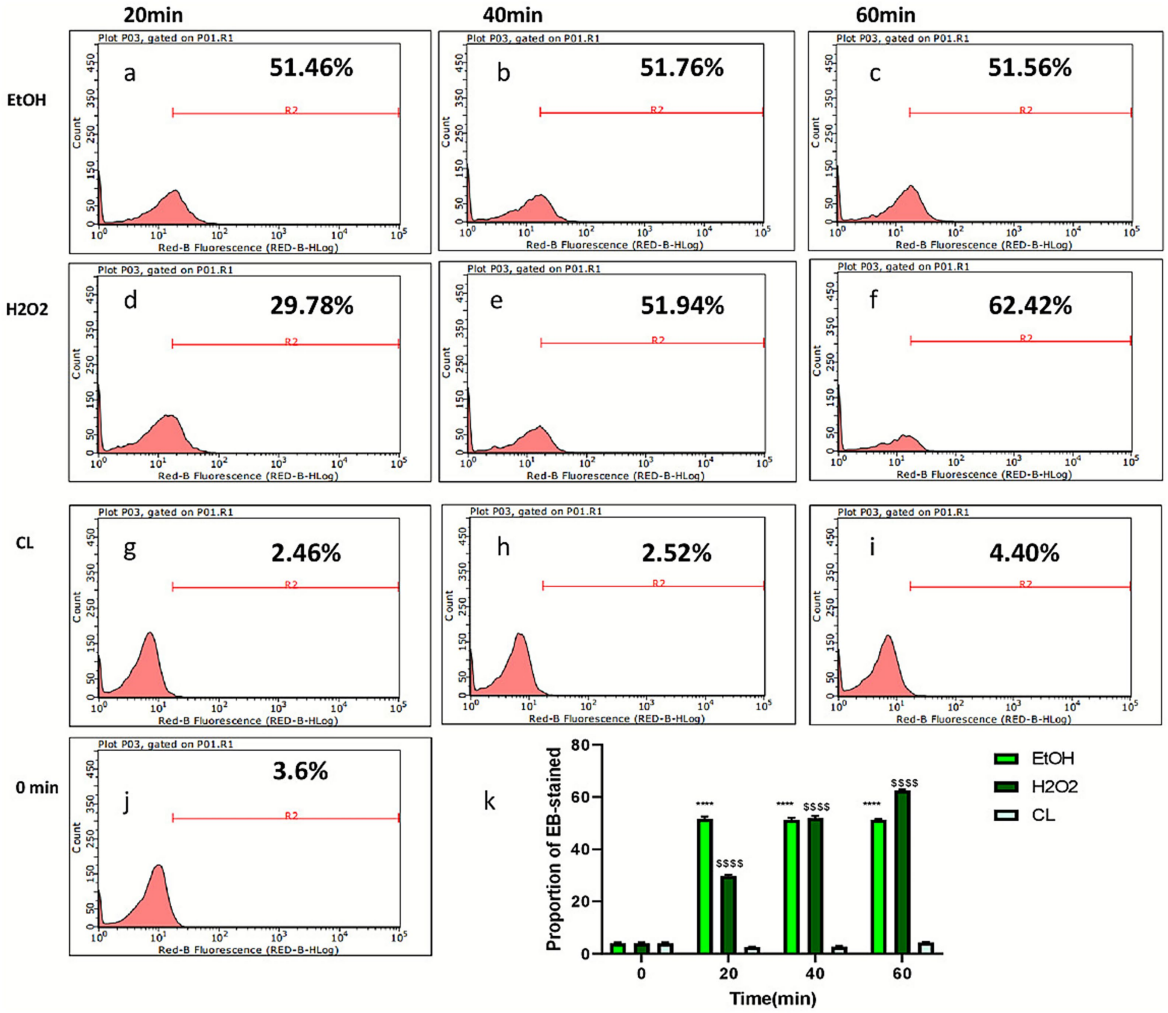
Flow cytometric analysis of EB staining in *Encephalitozoon cuniculi* spores following treatment with fast-acting disinfectants. **(a–c)** Show the flow cytometric results of EB staining in spores after 20, 40, and 60 min disinfection with 75% ethanol, respectively. **(d–f)** Represent the results following 20, 40, and 60 min treatments with 1% hydrogen peroxide. **(g–i)** Display the outcomes after 20, 40, and 60 min exposures to chlorine-based disinfectant. **(j)** Shows the untreated controls. **(k)** Presents statistical analysis of the proportions of EB-positive spores across disinfectants and time points. Data are expressed as mean ± SD, *n* = 3. ****p < 0.0001 vs. their respective untreated controls (Group 0).

Following the determination of the optimal EB staining concentration, we evaluated three fast-acting disinfectants: 75% ethanol, 1% hydrogen peroxide, and a chlorine-based disinfectant. Disinfection time points were set at 20, 40, and 60 min. Results for 75% ethanol demonstrated a statistically significant increase in the proportion of EB-positive spores compared to untreated controls at 20 min (*p* < 0.0001). Prolonged exposure beyond 20 min did not further enhance staining efficacy. Notably, during post-treatment centrifugation, the 75% ethanol group exhibited markedly whiter and larger spore pellets—a phenomenon absent in the other disinfectant groups. The chlorine-based disinfectant failed to exhibit significant differences in EB-staining proportions compared to controls across all time points, suggesting ineffective disruption of spore wall integrity. Among the tested agents, hydrogen peroxide showed the most substantial time-dependent increase in EB-positive rates, with 60-min exposure achieving a maximum staining proportion of 62.42 ± 0.34% (Figure 2).

**Figure 3:**
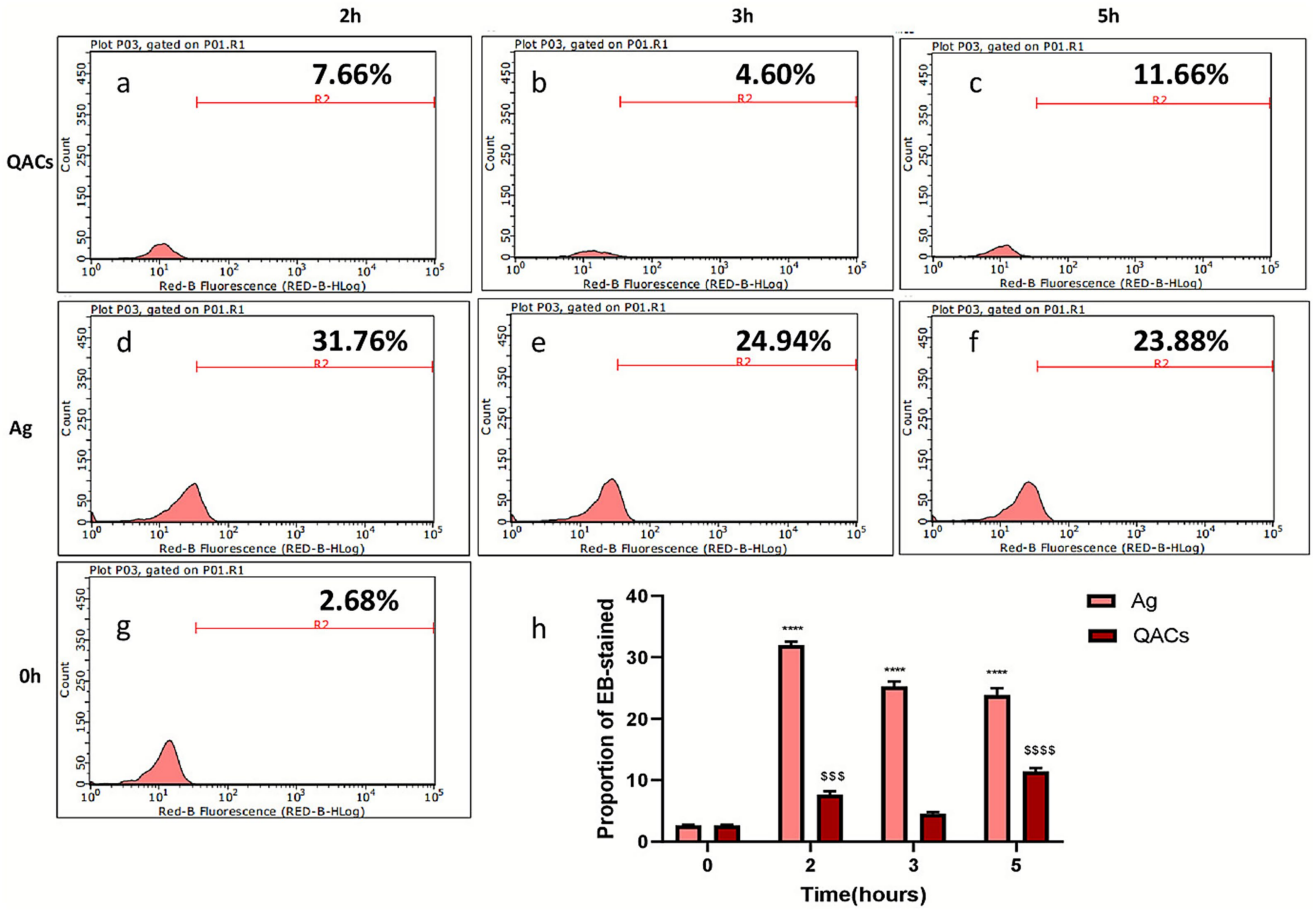
Flow cytometric analysis of EB staining in *E. cuniculi* spores following treatment with long-acting disinfectants. **(a–c)** Show the flow cytometric results of EB staining in spores after 2, 3, and 5 h disinfection with quaternary ammonium compounds (QACs), respectively. **(d–f)** Represent the results following 2, 3, and 5 h treatments with silver ion agents. **(g)** Shows the untreated controls. **(h)** Presents statistical analysis of the proportions of EB-positive spores across disinfectants and time points. Data are expressed as mean ± SD, *n* = 3. ****p < 0.0001 vs. their respective untreated controls (Group 0).

Subsequently, we evaluated two long-acting disinfectants, quaternary ammonium compounds (QACs) and silver ion agents, using the same methods as described above. Compared to rapid-acting disinfectants, the maximum EB-staining rate observed among long-acting agents was 31.76 ± 0.45% in the silver ion group. Notably, post-disinfection centrifugation of QAC-treated samples yielded persistently aggregated pellets that resisted resuspension, with spores forming compact clumps. This aggregation phenomenon potentially compromises the objectivity of flow cytometric assessment for QAC efficacy, as evidenced by characteristic shifts in forward scatter (FSC)/side scatter (SSC) profiles (Figure 3).

### 3.3 High-resolution qPCR monitoring of sporicidal effects on Sporozoan cellular infectivity

To accurately determine whether disinfectant-treated spores retained infectivity, we inoculated Vero cells with spores exposed to various disinfectants. Experiments were conducted in 12-well plates pre-seeded with synchronized Vero cells 24 h prior to inoculation. After 48-h infection, supernatants were meticulously aspirated to eliminate residual extracellular spores, followed by three PBS washes. Cells were lysed thoroughly, and the resultant lysates served as templates for *E. cuniculi*-specific qPCR. This protocol ensured the exclusive detection of intracellular spores, reflecting functional infectivity. Our findings demonstrated that chlorine-based disinfectants achieved superior infectivity ablation, with > 90% spore infectivity loss within 20 min. Although hydrogen peroxide showed slightly reduced efficacy compared to chlorine-based agents, it eliminated 96% of infectivity after 60-min exposure. In contrast, 75% ethanol (EtOH) exhibited minimal infectivity inactivation, yet showed statistically significant differences from untreated controls at all timepoints (*p* < 0.001).

Disinfectant efficacy was evaluated using the Log Reduction Value (LRV), defined as the log₁₀ reduction in the mean spore DNA copy number concentration from the untreated control to the disinfected sample. A larger LRV indicates superior efficacy (Rutala et al., 2023). The LRV is formally defined by the equation: LRV = N₀ − Nₓ, where N₀ and Nₓ are the log₁₀-transformed mean spore DNA copy number concentrations for the untreated control and disinfected groups, respectively (Rose et al., 2005; Huang et al., 2022). Consequently, when the post-disinfection mean concentration falls below 1 (making Nₓ ≤ 0), the calculated LRV will be greater than or equal to N₀ (Table 3).

**Table 3:** Intracellular *E. cuniculi* spore DNA copy concentrations across disinfectant treatment groups (mean ± SEM, *n* = 3).

Groups	Replicates	Copy concentrations	Log reduction value (LRV)
Control	3	7.054E3 ± 1.271E1	/
CL-20	3	4.068E2 ± 3.067E0	1.239
CL-40	3	N.D.	≥3.848
CL-60	3	N.D.	≥3.848
EtOH-20	3	4.923E3 ± 3.915E1	0.1562
EtOH-40	3	4.811E3 ± 1.381E1	0.1662
EtOH-60	3	4.879E3 ± 2.614E1	0.1601
H_2_O_2_–20	3	3.565E3 ± 6.424E0	0.2964
H_2_O_2_–40	3	1.740E3 ± 4.782E0	0.6079
H_2_O_2_–60	3	2.816E2 ± 2.209E0	1.399
Ag-2	3	5.277E3 ± 7.600E-1	0.1261
Ag-3	3	4.044E3 ± 9.912E0	0.2416
Ag-5	3	1.689E3 ± 5.388E0	0.6208
QACs-2	3	2.685E3 ± 1.644E1	0.4195
QACs-3	3	2.534E3 ± 1.310E1	0.4446
QACs-5	3	2.421E3 ± 1.644E1	0.4644
BLANK	3	N.D.	/

SEM, Standard Error of the Mean; N. D, Not detected; Log Reduction Value (LRV) was defined as LRV = N_0_–N_X_.

### 3.4 Fluorescent staining of vero cells following infection with disinfectant-treated *Encephalitozoon cuniculi*

**Figure 4:**
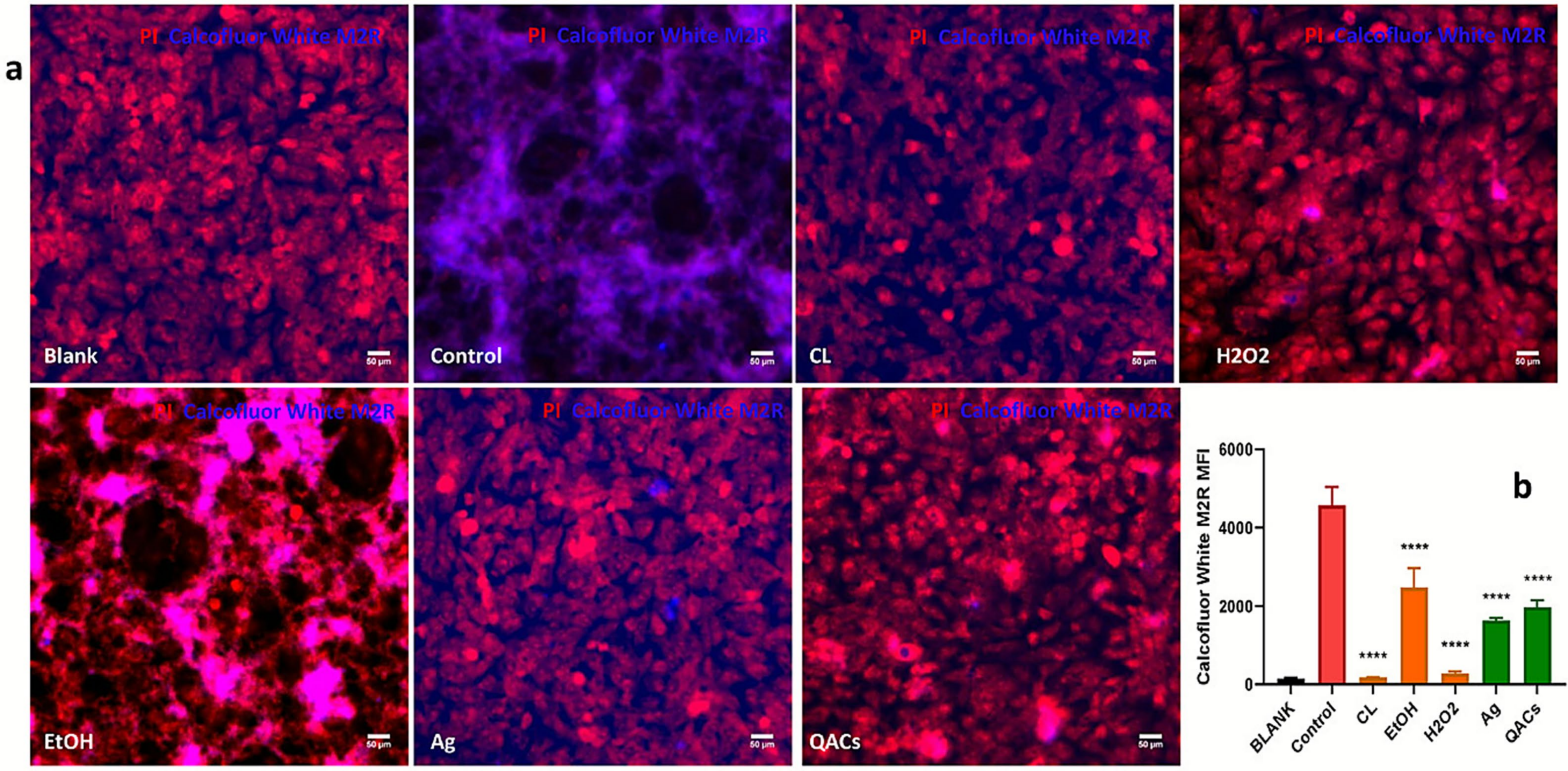
Fluorescent microscopy analysis of Vero cells infected with *E. cuniculi* spores exposed to various disinfectant regimens. **(a)** Merged images of PI and Calcofluor White M2R double-staining across different disinfectant treatment groups. **(b)** Quantitative analysis of fluorescence intensity derived from Calcofluor White M2R staining in each group. Fluorescence intensity was quantified using ImageJ software by measuring the mean fluorescence intensity of Calcofluor White M2R-stained signals from each image captured in the UV channel. Three independent imaging fields were analyzed per group. The control group consisted of cells infected with untreated microsporidia. Statistical analysis was performed using one-way ANOVA, with the mean fluorescence intensity of each treatment group compared to that of the control group. The symbol **** (*p* < 0.0001) denotes statistically significant differences between each disinfectant-treated group and the untreated control group. Groups showing statistically significant differences from the control are accordingly indicated in the figure.

To visually assess infectivity, spores subjected to various disinfectant treatments were recovered, washed three times with PBS, centrifuged, and finally resuspended in complete medium for cell infection.After 48 h, the supernatant was removed, and the cells were washed with PBS, followed by double staining with propidium iodide (PI) and Calcofluor White M2R—a dye that binds to polysaccharides such as cellulose (Green et al., 2000). Among the different treatment groups, the most intense Calcofluor White M2R staining was observed in the control group, followed by the ethanol group, while the chlorine-based disinfectant group exhibited the weakest staining (see Figure 4).

### 3.5 Quantitative assessment of Sporozoan burden in mice following sporicidal treatment

To validate the efficacy of disinfectants against *E. cuniculi in vivo*, spore suspensions were adjusted to 10^8^ spores per aliquot and treated with various long-acting and short-acting disinfectants. The control group underwent the same centrifugation and recovery procedures but without any disinfectant exposure. All mice were euthanized 45 days post-intraperitoneal injection, and blood and kidney tissues were collected for *E. cuniculi* DNA detection (Sak et al., 2020). Untreated control mice showed *E. cuniculi* DNA levels of approximately 10^4^ copies/μL in both kidneys and blood. In contrast, all disinfectant-treated groups exhibited markedly reduced or undetectable levels of *E. cuniculi* DNA. Notably, no *E. cuniculi* DNA was detected in either blood or kidney samples from mice exposed to the long-acting quaternary ammonium compound (QACs, 5-h treatment) or the short-acting disinfectants (chlorine-based agent and hydrogen peroxide, 1-h treatment). Conversely, 75% ethanol demonstrated the least efficacy among the tested disinfectants, with detectable *E. cuniculi* DNA persisting in both kidneys and blood of mice after either 20-min or 1-h treatments (Table 4).

**Table 4:** Quantification of *E. cuniculi* DNA in mouse kidney and blood after infection with differently treated spores (mean ± SEM, *n* = 3).

Groups	Replicates	Kidney copy concentrations	Log reduction value (LRV)	Blood copy concentrations	Log reduction value (LRV)
Control	3	4.948E4 ± 1.539E4	/	1.227E4 ± 3.61E3	/
CL-20	3	1.166E1 ± 8.96E0	3.628	2.33E0 ± 4.62E0	3.721
CL-60	3	N.D.	≥4.694	N.D.	≥4.089
EtOH-20	3	1.904E2 ± 2.043E1	2.415	1.95E0 ± 1.23E0	3.798
EtOH-60	3	8.335E1 ± 3.413E1	2.774	1.72E0 ± 2.55E0	3.853
H_2_O_2_–20	3	5.25E2 ± 2.055E2	1.974	1.14E0 ± 2.26E0	4.032
H_2_O_2_–60	3	N.D.	≥4.694	N.D.	≥4.089
Ag-2	3	1.863E2 ± 1.966E2	2.424	N.D.	≥4.089
Ag-5	3	3.597E1 ± 1.273E1	3.138	N.D.	≥4.089
QACs-2	3	1.53E0 ± 1E0	4.509	N.D.	≥4.089
QACs-5	3	N.D.	≥4.694	N.D.	≥4.089
BLANK	3	N.D.	/	N.D.	/

SEM, Standard Error of the Mean; N.D., Not detected; Log Reduction Value (LRV) was defined as LRV = N_0_–N_X_.

### 3.6 Fluorescence staining of kidney sections from mice in various infected groups following disinfection treatment

The kidney is a primary target organ of *E. cuniculi* infection (Rodríguez-Tovar et al., 2016). To corroborate the quantitative PCR (qPCR) results from disinfected tissue samples, kidney tissues from each experimental group were fixed, embedded, and sectioned. The rehydrated sections were subjected to fluorescence staining with Calcofluor White (CFW) and propidium iodide (PI). PI staining, which emits red fluorescence, was used to delineate tissue structure. Since the inner layer of the microsporidian spore wall is primarily composed of chitin, spores are an ideal target for CFW, which produces intense, specific blue–green fluorescence upon binding under ultraviolet light.

**Figure 5:**
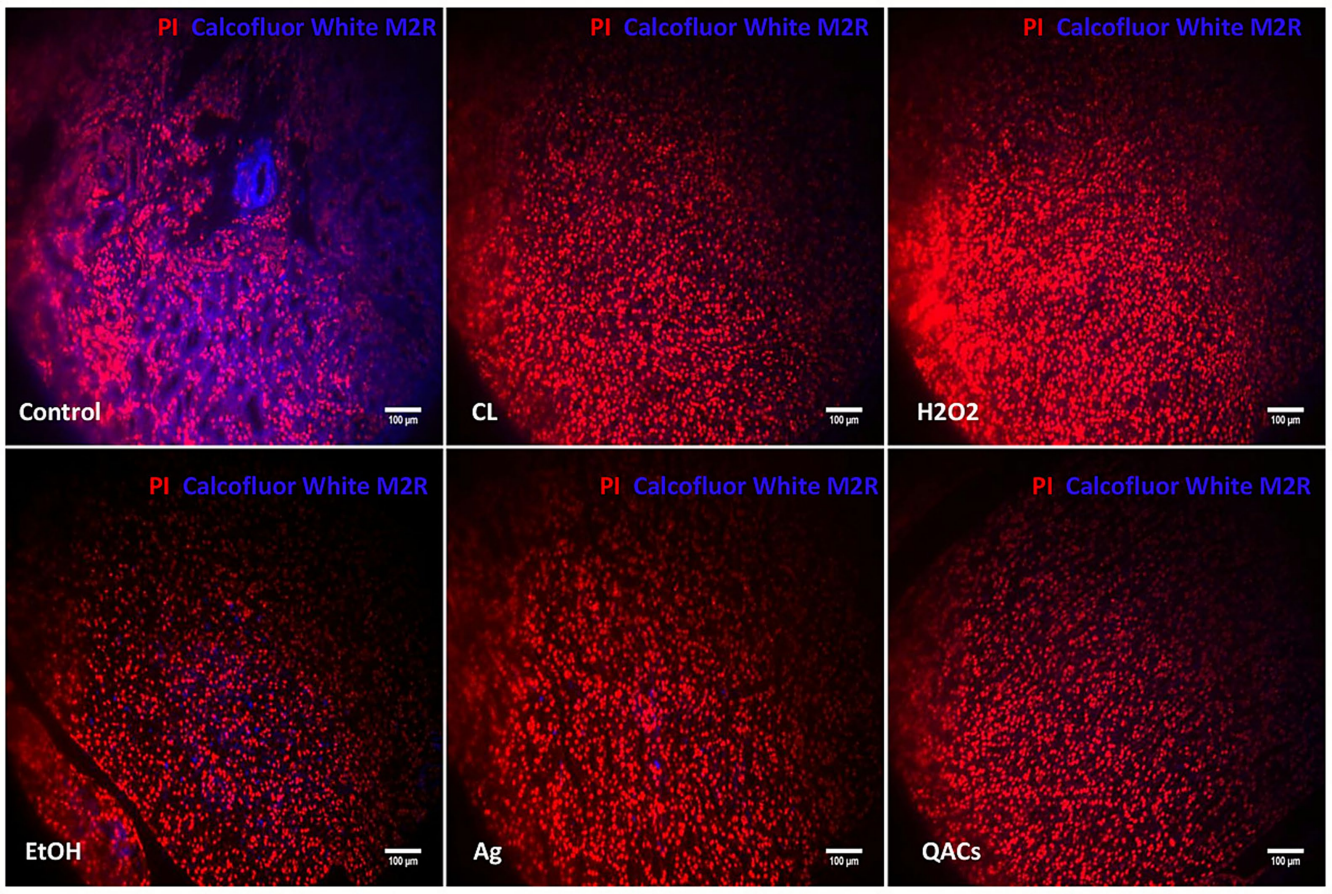
Representative fluorescence micrographs of mouse kidney sections dual-stained with Calcofluor White (CFW) and propidium iodide (PI). The untreated control showed intense staining, the EtOH and Ag groups displayed scattered patches, and all other disinfection groups (1-h rapid-acting or 5-h long-acting) showed absent staining. Images were taken at 200 × magnification (scale bar: 100 μm).

In the present study, kidney sections from untreated control mice exhibited strong blue CFW fluorescence signals, primarily localized to the walls of small blood vessels, the tubular basement membrane, and the pelvi-ureteric junction. In contrast, the CFW signal in kidney tissues from disinfectant-treated infected mice was markedly less intense and extensive compared to that in the control group. Notably, virtually no detectable CFW signal was observed in groups treated with the chlorine-based disinfectant for 1 h, hydrogen peroxide for 1 h, or the quaternary ammonium compound (QACs) for 5 h (Figure 5).

## 4 Discussion

This study provides a comprehensive, multi-modal evaluation of the sporicidal efficacy of various chemical disinfectants against *E. cuniculi*, an emerging microsporidian pathogen of significant public health concern (Han et al., 2019). By integrating flow cytometric analysis, cell-based infectivity assays, and *in vivo* murine infection models, we have systematically delineated the differential capacities of both rapid- and long-acting disinfectants to compromise spore structural integrity, abolish infectivity, and prevent systemic dissemination.

A key finding of our work is the pronounced divergence between spore membrane disruption—as assessed by ethidium bromide (EB) uptake—and actual infectivity abrogation. For instance, 75% ethanol induced substantial membrane damage (∼51.46% EB-positive spores within 20 min), yet failed to consistently eliminate intracellular invasion in Vero cells or prevent renal colonization in mice. This discrepancy underscores a critical limitation of structural integrity assays alone and highlights the necessity of complementing such methods with functional infectivity readouts. It further suggests that ethanol may compromise membrane permeability without fully inactivating the sporoplasm, permitting low-level but persistent infectivity. Our findings indicate that the commonly used 75% ethanol concentration, typically effective due to its permeabilizing and germicidal properties, demonstrates limited efficacy against *E. cuniculi* spores. EB staining confirmed this limited efficacy, showing that the proportion of stained spores plateaued at ~51% after 20 min of exposure. This suggests that a saturation point in membrane permeability may be reached, preventing further sporicidal action.In contrast, chlorine-based agents exhibited exceptional sporicidal activity, achieving near-complete inactivation of infectivity within 20 min and abolishing detectable *E. cuniculi* DNA in both cellular and murine models at 60 min. This aligns with prior reports on the oxidative potency of chlorine against microsporidian spores, though our data further refine the exposure thresholds required for reliable sterilization. Notably, hydrogen peroxide also demonstrated strong time-dependent efficacy, reaching > 96% infectivity reduction after 60 min, supporting its utility as a viable alternative in settings where chlorine residuals are undesirable.

Notably, the chlorine-based disinfectant, despite demonstrating superior efficacy in abolishing spore infectivity both *in vitro* and *in vivo*, exhibited a remarkably low level of EB staining across all timepoints. This apparent paradox—potent sporicidal activity without concomitant large-scale membrane disruption detectable by EB uptake—suggests a mechanism of action that bypasses gross structural disintegration (Young and Setlow, 2003). We postulate that chlorine, a potent oxidizing agent, may exert its primary effect through the selective oxidation of critical intramural and intracellular components rather than causing generalized membrane poration. Potential molecular targets include: (1) key enzymes within the spore’s germination apparatus (e.g., those in the polar tube extrusion machinery), whose inactivation would irrevocably block host cell invasion without immediate compromise of the spore wall’s barrier function against large molecules like EB; (2) specific structural proteins of the endospore layer, where cross-linking or oxidative damage could impair spore rigidity and germination dynamics while maintaining overall integrity against fluorescent dyes; and (3) direct oxidative damage to the spore’s genomic DNA, rendering it non-infectious even if the spore retains its structural semblance and dye-exclusion capacity. This hypothesis aligns with the established mode of action of chlorine against bacterial spores, where protein oxidation is a key event preceding the loss of viability. Consequently, while the EB staining assay is valuable for detecting overt membrane damage, it appears insufficient to capture the inactivation pathway employed by chlorine-based agents, which is functionally devastating yet structurally subtle. Our findings underscore the critical importance of coupling structural assays with functional infectivity readouts to fully evaluate sporicidal efficacy.

Among long-acting formulations, nano-silver and quaternary ammonium compounds (QACs) showed progressive sporicidal effects, with nano-silver achieving >76% infectivity reduction after 5 h. However, quaternary ammonium compound disinfectants can induce spore aggregation, which not only interferes with flow cytometry data analysis but may also compromise practical disinfection efficacy. This occurs because spore clustering creates a physical barrier effect: the outer layer of spores hinders disinfectant penetration, resulting in reduced effective concentration within the agglomerated clumps. Despite this, QACs achieved undetectable spore DNA levels in mouse models after prolonged exposure, affirming their potential for use in extended decontamination scenarios, such as veterinary kennels or hospital environments where continuous antimicrobial activity is advantageous.

The *in vivo* component of our study further validated the hierarchy of disinfectant efficacy observed *in vitro*. Mice challenged with spores treated with chlorine, hydrogen peroxide, or extended-duration quaternary ammonium compounds (QACs) showed no evidence of renal or bloodstream infection, whereas ethanol-treated spores led to measurable DNA loads in both compartments. These results not only corroborate cell-based findings but also emphasize the clinical relevance of adequate disposal duration and agent selection in preventing zoonotic transmission. From a methodological standpoint, our development and validation of a highly sensitive and specific TaqMan qPCR assay targeting the *E. cuniculi* ITS region enabled precise quantification across biological matrices, with a detection limit of 1.4 fg of genomic DNA. This represents a significant advancement over conventional staining or culture-based methods, which often lack the sensitivity to detect residual infectivity or low-level dissemination.

Notwithstanding these insights, our study has limitations. The influence of organic load—a well-known confounding factor in disinfection efficacy—was not evaluated, and future studies should incorporate realistic matrices such as urine or feces to better simulate environmental conditions. Additionally, variations in temperature, humidity, and spore age may alter disinfection kinetics and warrant further investigation.

In conclusion, our integrated experimental approach provides a robust evidentiary foundation for disinfection guidelines against *E. cuniculi*. We propose that chlorine-based agents and hydrogen peroxide be prioritized for rapid disinfection in high-risk clinical settings, while silver-ion and quaternary ammonium compound (QAC) formulations offer valuable sustained protection in endemic or high-contact environments. These findings not only address a critical gap in microsporidia infection control but also exemplify the imperative of linking structural, cellular, and whole-organism outcomes in the evaluation of antimicrobial agents.

## 5 Conclusion

In conclusion, this multi-modal investigation provides a rigorous, evidence-based hierarchy for the chemical disinfection of *E. cuniculi* spores. We definitively establish chlorine-based agents and hydrogen peroxide as the frontline choices for rapid and reliable spore inactivation in critical settings, capable of abolishing infectivity in both cellular and animal models within 1 h. Conversely, 75% ethanol, despite inducing measurable membrane permeability, demonstrated insufficient sporicidal efficacy, underscoring a critical risk if relied upon for microsporidia control.

A pivotal insight from our work is the dissociation between spore structural integrity and viability. This was exemplified by chlorine-based agents, which neutralized infectivity with exceptional efficiency without concomitant large-scale compromise of the spore wall to EB penetration. This finding underscores the limitation of relying solely on dye-exclusion assays and champions the necessity of functional infectivity assessments for definitive efficacy evaluation.

For scenarios demanding prolonged antimicrobial activity, nano-silver and quaternary ammonium compounds (QACs) present viable alternatives, achieving complete clearance of infection *in vivo* after extended exposure. By integrating molecular, cellular, and in vivo validation, this study transcends conventional disinfectant testing and delivers a robust, data-driven framework for developing infection control guidelines. Our findings offer critical insights for mitigating the transmission risk of this resilient microsporidian pathogen in healthcare, veterinary, and community settings.

## Data Availability

The original contributions presented in the study are included in the article/supplementary material, further inquiries can be directed to the corresponding author.
